# From concept to action: Operationalising animal welfare strategies in zoos and aquaria

**DOI:** 10.1017/awf.2025.10053

**Published:** 2025-12-12

**Authors:** Justine Kate Partoon, Sally Sherwen, Mark Ford Lester Smith, Lisa Riley, Alexandra Whittaker

**Affiliations:** 1 School of Animal & Veterinary Sciences, The University of Adelaide, Adelaide, SA 5371, Australia; 2 Zoos South Australia, Adelaide, SA 5000, Australia; 3 Wildlife Conservation and Science, Zoos Victoria, Melbourne, VIC 3052, Australia; 4 Faculty of Health and Wellbeing, University of Winchester, Sparkford Road, Winchester SO22 4NR, UK

**Keywords:** Animal welfare, aquarium, evidence-based welfare, positive welfare, strategy, zoo

## Abstract

Zoos and aquaria have an ethical responsibility to ensure the welfare of the animals in their care. Developing and implementing an animal welfare strategy is central to fulfilling this obligation. An animal welfare strategy is a comprehensive framework that integrates animal welfare into all zoo operations, policies, and procedures, aiming to embed effective animal welfare practices across the entire organisation and extend these practices into the broader community. The strategy should reflect a clear ongoing commitment to animal welfare, incorporate the latest developments in animal welfare science, ensure an evidence-based approach, and be fully integrated into all policies and procedures. In addition, the strategy should provide a clear framework, measurable goals, and key performance indicators (KPIs), to ensure a structured, objective approach to animal welfare monitoring and enhancement. Creating a strategy involves nine key steps. Structuring the strategy around these steps through the lens of four primary domains: animal care; animal welfare assessment; communication; and evaluation, ensures a comprehensive institution-wide commitment to animal welfare. Once established, the strategy should be sufficiently flexible to ensure continued self-examination and improvement, and an ability to incorporate key insights from the rapidly developing field of animal welfare science. Implementing such a strategy requires sustained effort, strong leadership, and an organisational culture that supports shared values and continual improvement.

## Introduction

Zoos and aquaria have undergone significant changes over time, shifting from their original role as places mainly focused on displaying animals for public amusement to becoming organisations dedicated to education, conservation, engaging the public, and advancing scientific knowledge (Miller *et al.* 2004; Barongi *et al.* 2015; Maynard *et al.* 2020). This shift in focus has led to a stronger emphasis on animal welfare. Zoos, whether large government-funded institutions, non-for-profit charities, privately owned facilities, or smaller wildlife parks, have an obligation to ensure the animals in their care experience the highest possible standards of animal welfare (Wolfensohn *et al.* 2018) reflecting ethical, legal, and professional considerations. Furthermore, accreditation by regional professional associations, such as the Association of Zoos and Aquariums (AZA), requires member institutions to meet established animal welfare criteria, and accreditation has been shown to correlate with improved compliance with animal welfare regulations when compared to non-accredited facilities (Riedman *et al.* 2023). While conservation goals are frequently central to a zoo’s mission, these are also expected to be pursued in ways that do not compromise animal welfare. Similarly, education and advocacy objectives are framed to support positive welfare outcomes. Therefore, it is essential that zoos have a mechanism to enhance positive welfare, where the absence of negative welfare is a given, whilst continuing to advance and adapt, aligning with changing public sentiment and new knowledge (Gray 2017). Placing animal welfare central to the zoo’s business, supports continuous improvement, and helps ensure the long-term success of zoos. Developing and implementing an animal welfare strategy can help achieve this objective.

In this work, we provide detail regarding the role and composition of an animal welfare strategy and aim to support readers in the creation and implementation of their own institutional animal welfare strategy. The framework presented here is aspirational and recognises that meaningful culture and operational change in zoos requires long-term commitment, leadership support and sustained collaboration. The paper first outlines the context within which an animal welfare strategy is created, by defining animal welfare and examining the societal and ethical considerations around the existence of zoos and then details the importance of good animal welfare for a zoo’s existence. It then provides a framework for developing and operationalising an animal welfare strategy to embed animal welfare into zoo policy and practice.

## Defining animal welfare

Defining animal welfare is important, as it shapes how zoos prioritise resources, communicate about animal welfare, and set organisational goals, supporting stakeholder ‘buy in’ and the strategic deployment of an animal welfare strategy. There have been various attempts to define animal welfare, but a central component of these definitions is that animal welfare is an internal state (Flavell *et al.* 2022) derived from the experiences of the animal as it interacts with the external environment. Animal welfare can vary on a scale from negative to positive and incorporates both physical and emotional health (Fraser 2008; Broom 2014; Yeates 2017). Many authors, including Fraser (2008), have argued the relative importance of three conceptual frameworks to ‘define’ animal welfare: (1) biological functioning; (2) naturalness; and (3) affective state. The term ‘affective state’ is often used to describe the emotions and moods experienced by an animal (Reimert *et al.* 2023), whether characterised as ‘pleasant’ or ‘unpleasant’ (Mellor & Beausoleil 2015).

Recent animal welfare research supports the central role of the affective state definition, which is centred on animal ‘feelings’, rather than focusing solely on biological functioning or naturalness (Duncan 2004; Whittaker & Barker 2020). Researchers in this camp argue that if an animal’s psychological needs are met, physical health and natural behaviour are likely to follow, making these other criteria less important to the definition of animal welfare (Englund & Cronin 2023). At its core, then, animal welfare is concerned with the individual’s internal state — their perception of their quality of life (Ohl & Putman 2018). One way of framing this approach to animal welfare is to summate the balance of positive and negative experiences throughout the animal’s life (Vigors *et al.* 2021).

Once a definition of animal welfare is settled upon, there needs to be some way of assessing it at a practical level. There have been various attempts to develop tools to assess animal welfare with the Five Domains model being commonly used in zoological parks, including in animal welfare assessment toolkits, developed by organisations such as the British and Irish Association of Zoos and Aquariums (BIAZA 2025) and the European Association of Zoos and Aquaria (EAZA 2025). This approach includes consideration of four physical domains: nutrition; environment; health; and behaviour, including behavioural interactions with conspecifics, humans, and the environment (Mellor *et al.* 2020). The fifth domain is the animal’s mental or affective state, evaluated by equating it with the collective influence of the four physical domains (Jones *et al.* 2022). The model considers both negative and positive contributions to the animal’s welfare state (Mellor *et al.* 2020). It is important to note that a thorough understanding of animal welfare science is fundamental to supporting an animal welfare strategy, as it influences how organisations define animal welfare, assess animal welfare, and set animal welfare goals.

## The social licence to operate

Animal welfare reflects not only the well-being of the individual animal, but also the ethical responsibilities of their caretakers, the mission of the institution, and the expectations of the broader community, with implications for institutional sustainability (Fraser 2008; Villarroya *et al.* 2024). Public perceptions of animal welfare play a central role in shaping the legitimacy and acceptance of zoological institutions, directly influencing their ability to continue operating within the context of society’s expectations (Villarroya *et al.* 2024). Over the past century, the progressive development of national and international animal welfare legislation, and professional standards, has reflected an increasing societal sensitivity regarding the needs and treatment of animals (Gavinelli & Knypinska 2015; Sandøe *et al.* 2020; Villarroya *et al.* 2024). Animal welfare is of increasing and significant societal concern, with citizens choosing to support or withdraw from animal-related organisations, such as zoological parks, marine parks, or public aquaria (collectively referred to as ‘zoos’), based on their views of how animals are treated and/or the justification(s) for captivity. This tacit approval, or societal contract often referred to as the ‘social licence to operate’ (SLO), represents an ongoing informal approval granted by the community and other stakeholders, reflecting society’s attitudes toward an organisation’s practices (Hampton *et al.* 2020). Failure to meet the responsibilities tied to social licence can result in social discontent, increased chances of protest and/or litigation, and stricter regulations, all which can hinder the success of animal industries (Coleman 2018) and the delivery of their core missions.

## Ethical considerations for zoos

Whilst zoos can deliver many positive outcomes, they also face certain dilemmas and challenges. Accredited zoos and aquaria have evolved to prioritise animal welfare, conservation, education, and research (Miller *et al.* 2004; Barongi *et al.* 2015; Maynard *et al.* 2020). As Gray (2017; p iii) aptly puts it, “*Good zoos aren’t menageries anymore, they’re conservation centres*”. One of the many challenges facing zoos is to ensure that conservation programmes are effective, whilst maintaining positive animal welfare (Paquet & Darimont 2010). When standards of animal welfare in zoos are poor, and there is apparent misalignment with stated goals, the effectiveness of any conservation messaging is diminished, potentially undermining public support (Spooner *et al.* 2023). However, some facilities exist primarily to function as entertainment venues with limited regard for conservation (Spooner *et al.* 2023). With this spectrum of operation in mind, it is important to acknowledge that not all zoos can be classified as conservation-focused. This conclusion notwithstanding, these zoos still need to consider animal welfare as a priority given the impacts that poor public perception and loss of social licence may have on their business.

Another challenge is appropriate species choice for public display. Species plans vary between zoos based on their mission and business priorities. For example, a zoo may focus upon conservation and visitor experience while facing challenges associated with animal welfare, sustainability, and financial capacity to house species (Harley 2023). Additionally, charismatic mammal species are often of interest to visitors (Brereton & Brereton 2020). However, many of these species, such as carnivores, cetaceans, primates, and elephants, are commonly associated with poor welfare, evidenced by frequent display of stereotypical behaviours that have traditionally been perceived as indicators of compromised welfare (Clubb & Mason 2007; Veasey 2020). Further challenges to welfare can arise due to population management, since limited space, restrictions on animal transfers, and genetic management goals can lead to practices such as culling surplus animals or suppressing reproduction, each of which may conflict with animal welfare considerations (Minteer & Collins 2013; Powell & Ardaiolo 2016), or fly in the face of public opinion. An animal welfare strategy supports organisational commitments to addressing these dilemmas, ensuring they are managed soundly through science, and that decision-making is ethically robust and effective.

## Why do zoos need an animal welfare strategy?

The public expect zoos to maintain high standards of animal welfare, and most zoos recognise this responsibility, whilst also ensuring legal compliance and adherence to accreditation schemes, many of which are predominantly welfare-focused. A well-articulated and socialised animal welfare strategy is essential to meet these expectations and to ensure the zoo’s ongoing SLO. A key component of a successful animal welfare strategy is the intentional alignment of the organisation’s goals with positive welfare outcomes. This alignment should be established as a clear operational position accepted by all parties.

Zoos also need to fulfil mission-based goals (e.g. conservation outcomes, educational engagement), both within the facility and in society at large. These goals frequently enhance the experience of zoo visitors. However, visitor engagement and the SLO plummet if these activities result in compromised animal welfare. In general, positive visitor engagement serves as an indication of an organisation’s commitment to, and delivery of, positive animal welfare, underpinning the broader mission of the zoo. As has been observed, optimal animal welfare contributes directly to successful conservation efforts (Escobar-Ibarra *et al.* 2021).

Tensions frequently arise when ethical considerations and business objectives clash, especially within the context of the resource constraints (e.g. financial, physical) common to many zoos. In these cases, zoos may adopt cost-effective solutions to make up for resource shortfalls. For example, volunteers may be used (in place of paid, full-time staff) for educational experiences involving animals, without having the full suite of requisite animal-handling skills (Martin *et al.* 2024). Similarly, an animal may require more of a limited resource (e.g. a larger habitat to meet their spatial needs), but the resource cannot be provided as it is financially or physically prohibitive. In these cases, as Maple and Perdue (2013; p 167) note, “*the business strategy has become* [the *de facto*] *animal welfare strategy*”.

An animal welfare strategy should protect the animals from an inadequate business plan and provide clear direction to inform decisions. In the absence of an animal welfare strategy there is a risk that animal welfare may be compromised by activities intended to meet financial shortfalls (e.g. a high level of paid animal-visitor interactions), or where physical resources supplied to the animals are restricted to ‘meet budget’ (e.g. replacement of nutritionally appropriate food with lesser alternatives). The animal welfare strategy may mitigate such concerns. An effective animal welfare strategy establishes a framework where this tension is addressed at the planning stage, before an animal’s welfare is compromised. A comprehensive and effective animal welfare strategy informs the business plan at the outset, guiding appropriate resource allocation, professional development for staff, etc, and in the process increases overall operational efficiency.

## The foundations of an animal welfare strategy

To genuinely uphold their SLO and their animal welfare commitments, it is important for zoos to adopt a comprehensive strategy that integrates animal welfare across all aspects of their operation. To accomplish this outcome, we propose a structured, step-by-step approach to developing an animal welfare strategy, tailored to meet the many needs of zoos. As described by More (2019), animal welfare policies are essentially ‘plans of action’. These policies should extend beyond the domain of animal husbandry to influence broader zoo operations, including conservation, education, financial planning, commercial operations, marketing, and more. By embedding animal welfare into all aspects of management, zoos can not only achieve enhanced animal care and welfare but also yield significant operational advantages and improvements to mission outcomes. These benefits include, amongst others, improved efficiencies such as better staff deployment, stronger community engagement, and increased public support.

Effective animal welfare strategies enable zoos to be progressive and proactive in achieving optimal animal welfare standards and ideally should reflect a clear commitment to applied animal welfare research, individual animal welfare monitoring, regular assessment of animal welfare, and ongoing improvements in support of positive animal welfare outcomes (Sherwen *et al.* 2018). In addition, a strategy should ensure that the zoo proactively tackles public concerns about animal welfare by conducting regular horizon scans (i.e. community sentiment research) to identify any areas that are uncomfortable for local communities, addressing challenges as soon as they are presented (Veasey 2022), and by directly advocating for improved animal welfare within the community. An animal welfare strategy should extend beyond simply mitigating animal welfare concerns, it should also establish a clear framework for actively enhancing and celebrating positive animal welfare.

**Figure 1.**
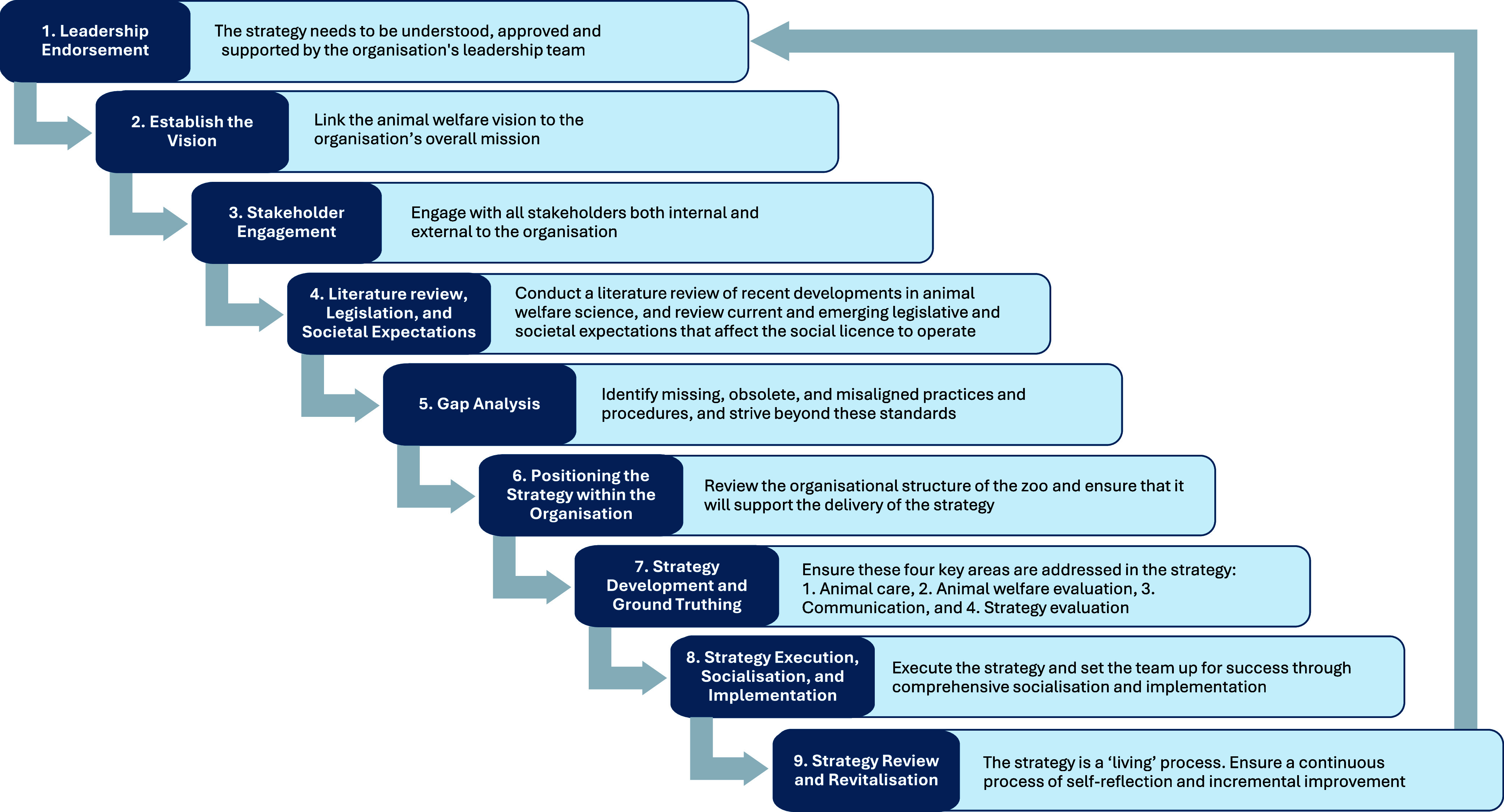
The steps involved in creating an animal welfare strategy for a zoo, showing key considerations.

Despite this clear understanding, there is little guidance available regarding how to create a zoo animal welfare strategy and the considerations upon which it should be based. A number of regional associations have created animal welfare strategies or frameworks (i.e. the World Association of Zoos and Aquariums’ ‘*Caring for Wildlife – Animal Welfare Strategy*’, with a new strategy being released in 2025, the Association of Zoos and Aquariums’ ‘*Strategic Framework for the Wellbeing of Animals*’ and the associated ‘*Guiding Principles*’ document, the British and Irish Association of Zoos and Aquariums’ ‘*BIAZA Welfare Policy*’, and the Zoo and Aquarium Association of Australasia’s ‘*Animal Welfare Policy*’). These documents provide useful guidelines and general principles for zoos to consider; however, they do not meet the needs of the local conditions unique to each zoo. It is important for individual zoos to develop their own strategy that links to regional or global networks, while also setting their own standards to make it relevant to their own community and organisational priorities. We set out the steps required to develop an animal welfare strategy for a zoo or aquarium in Figure 1, accommodating these specific conditions. This framework is intended as a flexible guide that can be adapted to suit the unique structure, priorities, and resources of individual organisations. Each step will be considered in turn, but it is important to note how integrated and interconnected each of these steps is when developing an animal welfare strategy.

### Step 1: Leadership endorsement

To ensure the success of a strategy, the policy-making process should be shaped by organisational culture, rules, and regulations, while also being informed by factors such as resource limitations (More 2019), so they can be overcome without animal welfare being compromised. A successful strategy requires backing from a coalition of key internal and external stakeholders, both at the time of its inception and adoption, and throughout its implementation (Crosby & Bryson 2018). A robust animal welfare strategy must be understood, approved, supported, and championed, by the organisation’s leadership team. While not all readers will be positioned to implement these changes directly, early engagement with leadership and identification of internal champions are the critical first steps. Building a culture that supports animal welfare innovation will take time and persistence but securing early ‘buy in’ and endorsement of an animal welfare strategy provides the foundation for lasting institutional change, especially if the development of the strategy was not initiated by the leadership team at the outset.

### Step 2: Establishing the vision

A critical step in the development of a strategy is to establish a vision for animal welfare, requiring broad-ranging stakeholder engagement to capture diverse perspectives, address ethical considerations, and encourage ongoing broad adoption. Having a clear definition of animal welfare is essential, as it aligns efforts towards common goals, supporting establishment of the vision. The first round of stakeholder engagement should conclude by distilling down all the received feedback into a strong collective vision statement for animal welfare and establishing the high-level primary components of the strategy. Such a vision should be aspirational, focused upon what the organisation wants to achieve and work towards in the realm of animal welfare. Stakeholder engagement will continue throughout the process, but the vision statement becomes a guiding star to the ongoing development of the strategy.

### Step 3: Stakeholder engagement

The strategy needs to capture as many of the needs of all stakeholder groups as possible. For strategic priorities to appropriately reflect stakeholder values and expectations, a multi-stakeholder approach is more effective than engagement at the level of a single stakeholder (Britton *et al.* 2021; Tanaka & Tanaka 2022). This approach is especially important for an animal welfare strategy, as stakeholders may share views, or have strongly differing opinions, in relation to what constitutes positive animal welfare, and how best to achieve optimal animal welfare for animals (Muhammad *et al.* 2022). Understanding the dynamics of stakeholder networks is vital, as collaborative approaches enable leaders in animal welfare to foster productive partnerships and develop policies that address the nuanced needs for animals, caretakers, and broader society (Fernandes *et al.* 2019). By incorporating diverse perspectives, the zoo will benefit from constructive feedback and collaborative problem-solving, which yields an improved strategy and better animal welfare outcomes.

Within a zoo setting, it is crucial to include ‘hands on’ stakeholders, such as animal care staff, veterinarians, and other animal management professionals. However, stakeholder groups should also encompass the full spectrum of zoo staff, including management, horticulture, technical services, marketing, visitor services, education, conservation, human resources, leadership, and volunteers. Each of these groups represent different business interests and contribute different sets of expertise, making the final strategy more robust.

Engaging the public during the consultation phase is also important for ensuring transparency and fostering positive community relations. In addition to animal advocacy groups, community engagement can include zoo members, visitors, and local organisations, alongside government representatives, philanthropists, researchers, and regional accreditation bodies for collaboration avenues, ensuring a fully inclusive approach. Whilst it is important to engage all stakeholders, it is equally essential to recognise that not all stakeholders have an equal level of interest, knowledge, or influence (Verrinder & Phillips 2023) on the final strategy.

External and internal stakeholder feedback can be obtained in many ways, including surveys, workshops, focus groups, one-on-one interviews, literature reviews, and community engagement sessions. Collaborating with other zoos and aligning priorities is important, as it can yield a significant positive additive impact, as opposed to working against one another. Collecting feedback from all stakeholders, including regional accreditation bodies, encourages them to take responsibility for enhancing programme performance (Sheperis & Bayles 2022), ensuring the strategy remains relevant, dynamic, and responsive, and helps secure organisation- and community-wide support.

### Step 4: Literature review, legislation, and societal expectations

Conducting a literature review on current animal welfare science pertaining to animals housed in zoos will help establish a strong knowledge base, identify gaps in the strategy, and help to inform policy and procedures. An animal welfare strategy should consider regional zoo association animal welfare and accreditation standards, recent developments in our collective understanding of animal welfare science, relevant stakeholder feedback (discussed above), an understanding of the available institutional resources, and the zoo’s aspirational goals and core mission. To aid in this process, all existing relevant internal policies and procedures related to animal welfare should be reviewed and updated. This documentation can include strategic business plans, with associated key performance indicators, animal welfare charters and codes of conduct, animal record-keeping processes and archival record-keeping, husbandry manuals, ongoing research programmes, species management and planning processes, and the animal welfare and ethics committee’s terms of reference (see below). As part of this policy review process, it is critical to assess existing animal welfare evaluation mechanisms at the zoo, which could include animal welfare assessments, animal transfer protocols, enclosure design protocols, quality of life assessments, health and nutritional assessments, environmental enrichment programmes and protocols, and behavioural monitoring programmes (such as animal-visitor interactions and animal training). In addition, a review of municipal, state, and federal animal welfare legislation and accreditation processes is recommended, as they are the primary bodies responsible for defining, penalising, and ideally preventing animal cruelty (Morton *et al.* 2020), and promoting positive animal welfare.

### Step 5: Gap analysis

Once the aforementioned resources have been collected and compiled, a gap analysis should be performed. The gap analysis should identify missing, obsolete, or misaligned practices and procedures, ensuring that they align with legislation and best practice, noting that legislative requirements frequently are intended to prevent negative animal welfare states (Melfi 2009), and that best practice aims to strive beyond these standards. Evidence-based management, such as using behavioural data to guide animal management, has gained significant momentum in recent years (Kaufmann *et al.* 2019; Brereton & Rose 2022) and should be included in the gap analysis, as evidence plays a key role in inferring animal welfare states. The gap analysis is essential in highlighting discrepancies between aspiration and reality (Kaplan *et al.* 2008), setting the foundation for a more robust animal welfare strategy, and for more achievable and effective practices.

### Step 6: Positioning the strategy within the organisation

**Figure 2.**
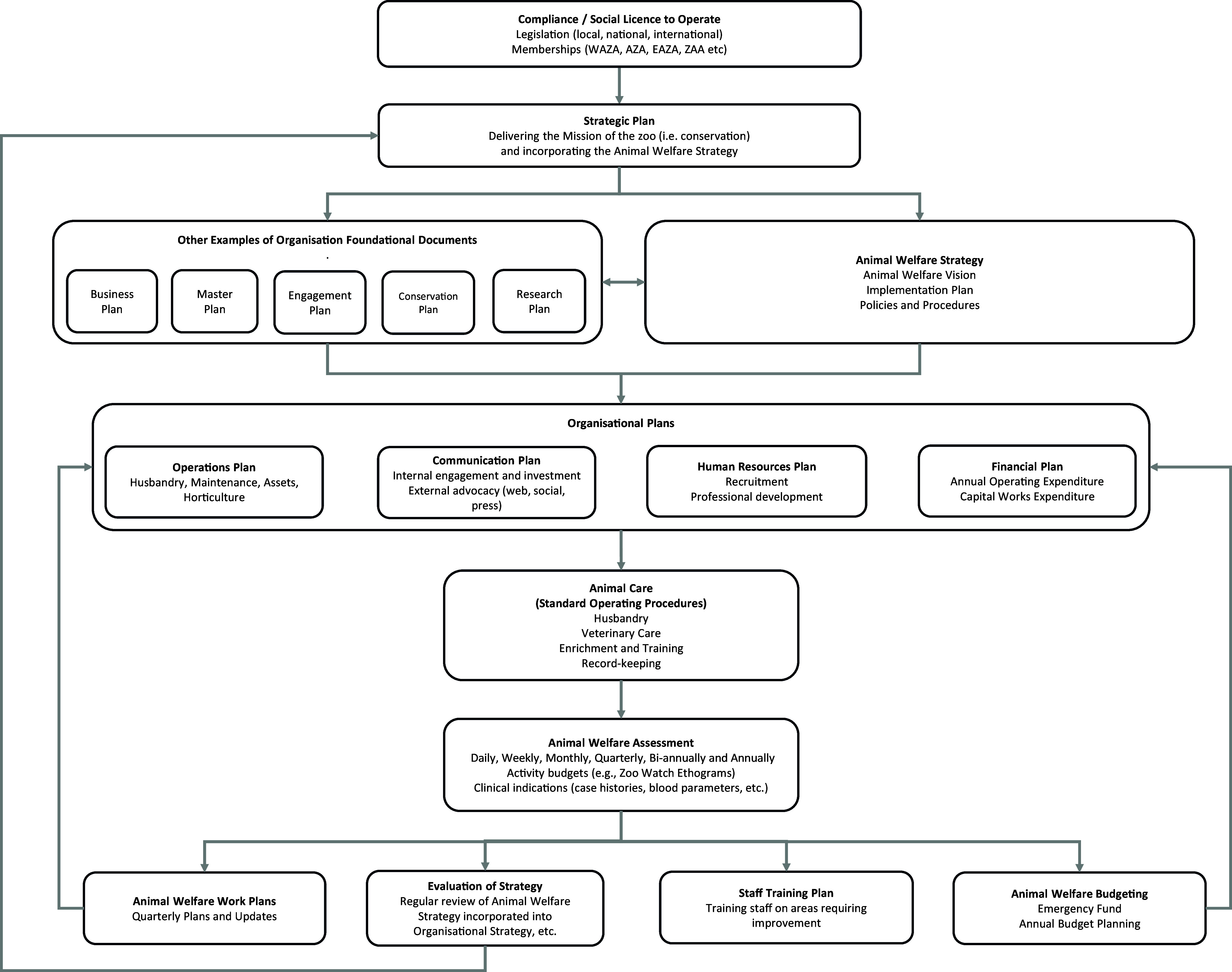
The operational ‘ecosystem’ of a modern zoo, showing the central role of the animal welfare strategy.

A crucial component of any animal welfare strategy is determining how it sits within the broader organisational structure of the zoo. Most zoos have a set of foundational documents that guide their operations, including planning documents (e.g. master plan, business plan) and operational charters (e.g. sustainability charter, accessibility charter). These guiding documents are typically reviewed and updated every three to five years, and are aligned under an overarching organisational strategic plan, intended to ensure the delivery of the organisation’s mission. To be most effective, an animal welfare strategy should be fully embedded within this interconnected business framework, particularly within the organisational strategic plan, to ensure that all guiding documents align and support one another (Figure 2).

The strategy should reference legislative and other compliance requirements and align with the organisation’s guiding principles and foundational documents, and in turn should inform institutional plans, e.g. operations, communication, human resources, financial, etc. These elements work together to deliver animal care and welfare outcomes, and incorporate the animal welfare evaluation process, which drives the creation of animal welfare work plans, staff training plans, and budgeting priorities. The organisational framework emphasises the importance of continuously evaluating the effectiveness of the animal welfare strategy to ensure ongoing improvement and adherence to the highest standards of animal welfare.

The next key step in advancing an animal welfare strategy is to establish a team responsible for developing and implementing policies and programmes aimed at understanding, improving, and evaluating animal welfare (Kagan *et al.* 2015). This team needs to take ownership of the strategy, be empowered to act under it, and to make improvements where needed. It is critical that this team is adequately resourced for the task. Whilst a team-based approach is ideal, the strategy still requires a ‘champion’, a person that is primarily responsible for managing the day-to-day planning process. The champion should track progress, push the process along, encourage forward momentum, and ensure all details are carefully attended to (Crosby & Bryson 2018). The champion must have the unequivocal support of the zoo leadership team. Some zoos employ dedicated animal welfare scientists, as recommended by Ward *et al.* (2018), who can promptly address early animal welfare challenges identified by keepers and veterinarians, conduct targeted research, and collaborate with external experts. Recruiting highly educated scientists skilled in research, public education, and responsible decision-making, is also beneficial, as their expertise, whether in-house or through strategic partnerships, helps prevent misdirected criticism and poor decision-making (Maple & Perdue 2013). The animal welfare champion should ideally be determined, well-versed in animal welfare science, and an inspiring communicator who can articulate a clear vision and ensure the successful development and implementation of the strategy.

### Step 7: Strategy development and ground truthing

To ensure the animal welfare strategy sets up an environment that effectively manages, assesses, and improves animal welfare it is helpful to break the strategy down into the following four components: (1) animal care; (2) animal welfare assessment; (3) communication (internal and external); and (4) strategy evaluation. Each of these components should be aligned with measurable goals and will be described in more detail below.

**Figure 3.**
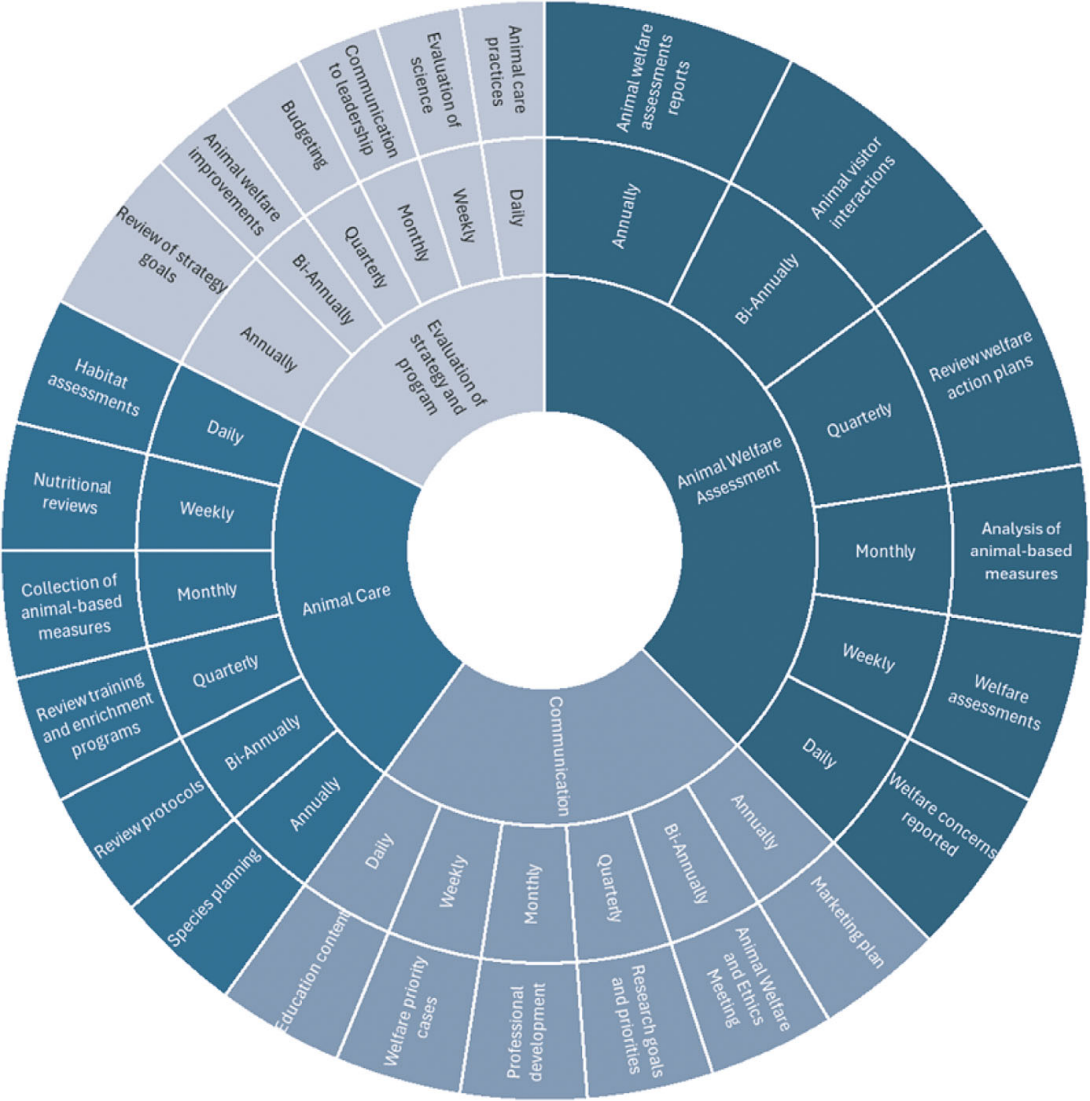
A schematic example of an Animal Welfare Strategy Framework for a zoo, including breakdown of KPIs by time-frame.

Once the framework is established, clear objectives should be defined using measurable, time-bound key performance indicators (KPIs) to track progress that aligns with the strategy and support the overall vision. A timeline with milestones and regular evaluations allows for continuous refinement. Strategic goals may be monitored and effectively met by aligning them with the specific, measurable, achievable, relevant and time-bound (SMART) model. The sunburst diagram detailed in Figure 3 illustrates an example of an evaluation process where KPIs are tracked at effective intervals. The suggested frequencies are intended as a guide rather than a rigid standard, and will naturally vary depending upon the size, resources, and staffing of each organisation. For example, well-resourced organisations may aim to complete one formal welfare assessment or nutritional review each week, while others may monitor these areas less frequently, focusing on key indicators such as body condition scoring or food intake as part of routine checks. The aim is to encourage regular, proactive monitoring of key areas at a pace and level of detail appropriate to each organisation, recognising that even incremental progress supports higher welfare standards. This structured approach ensures continuous monitoring across all aspects of the strategy, from animal care to staff training, fostering ongoing improvements in animal welfare standards.

#### 1. Animal care

Zookeepers play a critical role in providing care for animals (Sherwen & Hemsworth 2019). To achieve their goals, zoos should require that all staff responsible for animal care possess a strong understanding of recent advancements in animal welfare and husbandry techniques (Bacon *et al.* 2023). Yet, whilst there is a connection between animal care and animal welfare, they do not seamlessly align. Animal welfare indicators are typically divided into two primary categories: (1) resource-based indicators, or ‘inputs’; and (2) animal-based measures, or ‘outputs’. Inputs refer to the resources or management practices provided to the animals, such as space, environmental complexity, healthcare, and nutrition; and outputs assess the animal’s actual welfare state through a variety of means, for example, physiological health and behavioural indicators (Velarde & Dalmau 2012; Tallo-Parra *et al.* 2023). Both inputs and outputs are important to understand and quantify when assessing animal welfare.

This paper is not intended to detail every aspect of animal care for different zoo species. That work is for the relevant taxon specialists and animal carers/keepers. However, when creating an animal welfare strategy, all animal care disciplines need to be considered, including animal training and behaviour management programmes, enrichment programmes, nutrition evaluation and food preparation, healthcare, habitat design and substrate selection, animal visitor interaction programmes, and professional development for staff. Each of these disciplines implies inputs that must be examined when assessing animal welfare.

Provision of adequate resources alone does not guarantee that an individual will experience positive animal welfare (Kagan *et al.* 2018). Animal-based measures, or outputs, must be assessed to fully understand the welfare status of the animal in question. Optimal animal welfare is only achieved when an animal’s physical, social, and psychological needs are fully met, including the essential ability to exercise agency, make decisions, and have control over their daily activities (Kagan & Veasey 2010). It is also important to remember that every individual is unique, resulting in individual-specific needs (Tallo-Para *et al.* 2023) and a broader range of possible collective experiences and responses to the environment.

#### 2. Animal welfare assessment

Evaluating animal welfare within a zoo can be challenging, as there is no universally standardised method, and many zoos lack the formalised procedures for animal welfare assessment. However, many well-managed zoos, along with regional zoo associations, have taken proactive steps to develop their own systems for assessing, evaluating, and enhancing animal welfare. These assessments are often informed by standards, regulations, and guidelines from accreditation bodies such as the Association of Zoos and Aquariums (AZA 2025), the Zoo and Aquarium Association Australasia (ZAA 2025), the World Association of Zoos and Aquariums (WAZA 2025), as well as BIAZA (2025) and EAZA (2025).

Effective animal welfare evaluation requires the use of clear, objective, evidence-based assessment methods. These assessment tools can be developed internally and can include species-specific or taxa-specific tools for different animal groups (e.g. birds, mammals, reptiles, ungulates). Many zoos have already developed these tools and are more than willing to share with others, which can be a great place to start and can save significant time, for example, using the EAZA Welfare Assessment Library, where institutions like Wild Planet Trust have shared useful examples. Animal welfare assessments should extend beyond merely vertebrates (Escobar-Ibarra *et al.* 2021), as zoos have an ethical responsibility to promote good animal welfare for all individuals and ensure that animals, no matter their taxon, are experiencing optimal welfare. In addition, assessments may need to be tailored to accommodate different life stages or reproductive statuses. Recent progress in the field of animal welfare assessments has led to the development of various tools, including universal animal welfare framework surveys (Kagan *et al.* 2015), species-general animal welfare assessments (O’Brien & Cronin 2023), animal welfare risk assessments (Sherwen *et al.* 2018), and species-specific evaluation methods (Clegg *et al.* 2015; Benn *et al.* 2019).

Many tools use behaviour analyses as a source of evidence when assessing animal welfare. Monitoring animal behaviour is crucial, as it provides insight into the emotional states of individuals (Bacon 2018; Wolfensohn *et al.* 2018), and the animals’ responses to their surroundings, allowing an inference of animal welfare state. Despite the clear utility of behaviour as a measurable output, the reader is cautioned against over-reliance upon this metric alone, as it can bias and compromise the assessment. In addition, other animal-based measures, such as physiological indications, keeper ratings (Tallo-Parra *et al.* 2023), and qualitative behavioural assessments (Yon *et al.* 2019), as well as cognitive bias (Clegg 2018) analyses, can be applied as an adjunct. Readers seeking a more detailed exploration of this matter are encouraged to consult Jones *et al.* (2022) and Tallo-Parra *et al.* (2023).

#### 3. Communication (internal and external)

Communication of the animal welfare strategy, in a coherent and consistent manner, is fundamental to its successful implementation. This process should include both internal and external stakeholders to ensure broad awareness of the strategy, and clarity regarding the required actions to support its implementation. The key elements to consider for inclusion in a communication section of a strategy include establishment of an animal welfare and ethics committee, an animal welfare and ethics charter, education/professional development for staff, a social media policy, mechanisms for visitor engagement with animal welfare, and an animal welfare feedback process.

If it does not already exist, it is highly recommended to establish an animal welfare and ethics committee, including different departments within the zoo and some independent non-zoo members with knowledge of animal welfare science (e.g. a staff member of the Royal Society for the Prevention of Cruelty to Animals [RSPCA]). This committee plays an important role in overseeing the animal welfare strategy, ensuring the ethical treatment and welfare of animals throughout the organisation. The committee can provide independent advice on animal welfare questions, ethical dilemmas or ‘wicked’ problems, compliance, community engagement, and relevant animal welfare research. The committee should be composed of staff members who are well-versed and trained in animal welfare science, and internal and external stakeholders with and without formal training in animal welfare to support broader objectivity (Miller & Chinnadurai 2023).

In addition to the establishment of a dedicated committee, an animal welfare and ethics charter should be drafted and implemented that clearly articulates the zoo’s expectations about how its staff will engage with animal welfare throughout their day-to-day operations. A charter is stronger if the staff are mandated to read and commit to the charter. Furthermore, it is recommended that the zoo’s mission statement clearly and transparently prioritises animal care and welfare, positioning them as central to its values, akin to the importance of conservation in a modern zoo (Maple & Perdue 2013).

Another key aspect of a comprehensive animal welfare strategy is educating stakeholders about animal welfare science. Brando *et al.* (2023) found that many zoo professionals do not have access to appropriate professional development opportunities. Educating stakeholders is essential, as assessing animal welfare is seemingly intuitive and yet it is a nuanced and complex matter with significant societal implications. Passionate disagreements can ensue when stakeholders have different perspectives on how animal welfare should be defined and evaluated (DiVincenti *et al.* 2023). Educating zoo staff and providing them professional development opportunities, such as conference attendance, animal welfare courses, assistance with accreditation, etc, empowers them to apply scientific principles to questions of animal welfare, ensuring decisions are guided by evidence and best practice and not by visceral reactions and intuitions.

Social media continues to grow in popularity and is an effective platform for sharing information rapidly and to a very large audience (Meikle 2016), so it can be a powerful communication tool. It is highly recommended that, if not already in existence, the zoo adopt a clear social media policy to effectively communicate and support their commitment to positive animal welfare and transparency that is aligned with the zoos’ mission. For example, the policy could state that disseminating animal media only occurs when there is a clear purpose, e.g. a veterinary procedure in the event that an animal’s welfare is compromised, and where no additional supportive text is needed to clarify the nature of the procedure.

**Figure 4.**
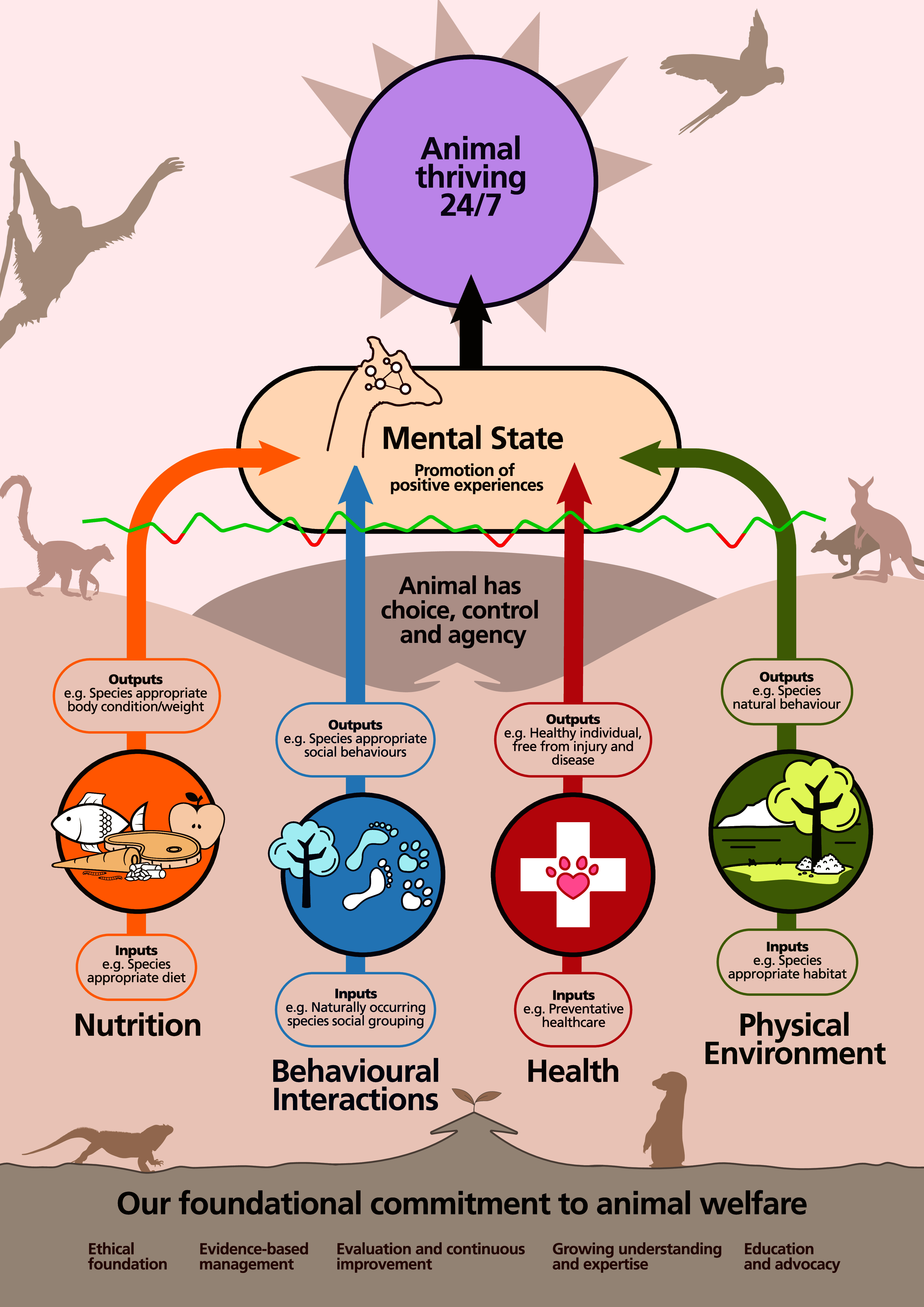
Infographic illustrating the Five Domains model of animal welfare applied to zoos, highlighting both input and output measures to help staff and visitors understand the key components of animal welfare assessment. Figure created by JKP and reproduced with the permission of Zoos South Australia.

It is important that visitors develop an understanding of animal welfare when visiting zoos. Figure 4 presents an example infographic used to communicate the Five Domains model of animal welfare as it applies to zoo animals. This visual overview makes it easier for both staff and the public, especially those who do not work directly with animals, to understand the key components of the Five Domains model and associated techniques used to manage animal welfare. By highlighting both input and output measures for assessing animal welfare, the infographic serves as an educational tool, promoting awareness and fostering better communication about how animal welfare is monitored and maintained. This visual guide helps demystify animal welfare, encourages a deeper understanding of, and connection to, the zoo’s mission, and empowers staff and visitors alike to contribute to the overall welfare of animals.

One final and essential form of communication must be included within the strategy: a mechanism that allows staff, volunteers, or zoo visitors to raise questions or concerns regarding animal welfare without fear of censure, retribution, or derision. A well-functioning team escalates concerns about animal welfare up through identified channels within the organisational hierarchy, with a formal response provided once the concern has been investigated (Kagan *et al.* 2015). In rare circumstances, where this approach is not functioning, an alternative mechanism should be provided, such as an anonymised email inbox. This approach can entail an in-confidence, direct communication with the animal welfare officer or the animal welfare and ethics committee. In addition, the culture surrounding what is traditionally considered ‘whistleblowing’ needs to change, so that reports of animal welfare concerns are viewed as valuable ‘information gathering’. Zoos should embrace open feedback as an opportunity to enhance transparency and improve their practices, rather than something to be resisted.

#### 4. Strategy evaluation: A continuous journey of improvement

Strategy evaluation is an ongoing process. Tracking KPIs, and assessing longer-term metrics, that indicate the strategy is working or not working, can include measures, such as changing trends in animal welfare evaluation scores, the range and effectiveness of enrichment programmes, the proportion of animals in preventative healthcare training programmes, or the extent to which real-time animal welfare tracking is used across the zoo, to name just a few.

In addition to specific and directed metrics, it is valuable to evaluate broader measures of the zoo’s performance. Financial support tied directly to animal welfare is a measure of success, as is the proportion of staff undertaking animal welfare professional development, both of which build capacity for improving animal welfare standards. Feedback from stakeholders, both internal and external, is another measure of strategy evaluation. Gathering and responding to these insights ensures that the strategy remains aligned with the needs and expectations of the community. Finally, ground truthing the strategy, with direct evidence, ensures it delivers on its promise of improving animal welfare.

### Step 8: Strategy execution, socialisation, and implementation

Once the strategy has been developed and mechanisms are in place to check its effectiveness, it is time to execute. Organisational changes often fail when improvements to practices and procedures are not implemented effectively (Tuite *et al.* 2022). Successfully executing a strategy requires the ability to effectively implement the planned actions and achieve the outlined goals (Abedian & Hejazi 2023). Implementing an animal welfare strategy effectively depends upon ensuring that all staff members have a shared and comprehensive understanding of its structure, goals, and objectives. Maintaining a flexible approach, whereby KPIs are regularly reviewed and adjusted as needed, ensures the strategy remains responsive to emerging challenges and helps staff stay focused and motivated. While focusing on opportunities for improvement is important, it is equally essential to celebrate success and acknowledge progress made towards improved animal welfare, recognising that, like any definition of animal welfare, the focus should not only be on addressing compromised animal welfare but also on proactively enhancing positive animal welfare outcomes.

### Step 9: Strategy review and revitalisation

For an animal welfare strategy to be truly effective, it must go far beyond a simple ‘tick box’ exercise. An effective strategy requires strong support from leadership and management, at all levels, to ensure that it is integrated into the zoo’s core operations and fully supports the zoo’s overarching mission. Effective evaluation goes beyond tracking data and outcomes, it should also include an ongoing cycle of reflection and adaptation. This approach requires strong support from leadership and management, at all levels, to ensure that it is integrated into the zoo’s core operations and fully supports the zoo’s overarching mission. It is essential to monitor not simply successes but also instances where expectations have not been met. Identifying deviations from expected outcomes, or areas where the strategy did not deliver as anticipated, can highlight areas that need more attention, adjustment, or resourcing. In addition, it is critically important that the zoo’s team remain informed of the latest developments in animal welfare science and incorporate changes to best practices wherever they emerge. This approach ensures that the zoo in question is able to continually improve its animal care practices and the associated animal welfare outcomes. The animal welfare and ethics committee is a good organising mechanism to keep the strategy on track and relevant and, ultimately, result in positive animal welfare within the zoo and the broader community at large.

## Conclusion

Zoos must adopt a comprehensive strategy to ensure the ongoing assessment and enhancement of animal welfare, rooted in the latest scientific advancements. Establishing a clear framework, endorsed by leadership and adequately resourced, provides a strong foundation for a coherent strategy and improved animal welfare. An effective strategy should be structured around four primary areas: animal care; animal welfare assessment; communication; and strategy evaluation, ensuring a comprehensive institution-wide commitment to animal welfare. Meaningful stakeholder engagement is vital to embed animal welfare into policies, practices, and daily care, with a commitment to maintaining animal welfare throughout each animal’s lifetime. Transparency in evaluating and implementing animal welfare standards builds public trust and reinforces the zoo’s social licence to operate. Furthermore, strategic planning should cascade seamlessly from high-level objectives to operational practices, ensuring animal welfare is prioritised across all aspects of zoo management. By integrating these different elements, zoos can create a robust system that meets ethical and scientific standards, while fostering continual improvement in animal welfare.

Implementing an animal welfare strategy of this scope is expected to require sustained effort over several years, guided by committed leadership and shared values across the organisation. Over time, this sustained approach strengthens institutional culture, ensures that animal welfare remains central to decision-making, and supports public trust and social licence. By maintaining a strategic, adaptive, and evidence-informed approach, zoos can embed welfare-focused values into all levels of operation and secure lasting benefits for both animals and the organisation.
